# Integration Strategies and Formats in Field-Effect Transistor Chemo- and Biosensors: A Critical Review

**DOI:** 10.1021/acssensors.4c03633

**Published:** 2025-04-15

**Authors:** Željko Janićijević, Larysa Baraban

**Affiliations:** § Institute of Radiopharmaceutical Cancer Research, Helmholtz-Zentrum Dresden-Rossendorf e. V. (HZDR), 01328 Dresden, Germany; † Else Kröner-Fresenius Center for Digital Health (EKFZ), Technische Universität Dresden (TU Dresden), 01309 Dresden, Germany

**Keywords:** field-effect transistor (FET), integration, multiplexing, sensor arrays, chemical sensors, biosensors, electronics, microfluidics, multisensor systems

## Abstract

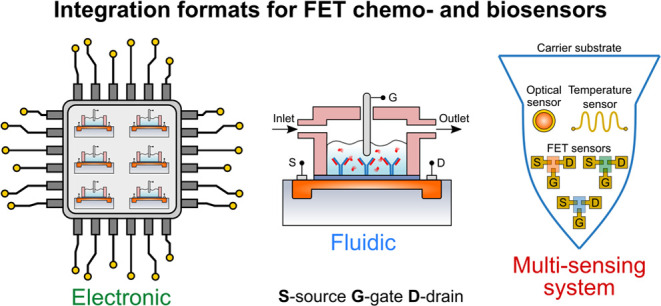

The continuous advances in micro- and nanofabrication technologies have inevitably led to major improvements in field-effect transistor (FET) design and architecture, significantly reducing the component footprint and enabling highly efficient integration into many electronic devices. Combined efforts in the areas of materials science, life sciences, and electronic engineering have unlocked opportunities to create ultrasensitive FET chemo- and biosensor devices that are coupled with more diverse and complex integration requirements in terms of hardware interfacing, reproducible functionality, and handling of analyte samples. Integration of FET chemo- and biosensors remains one of the major bottlenecks in bridging the gap between fundamental research concepts and commercial sensing devices. In this review, we critically discuss different strategies and formats of integration in the context of key requirements, fabrication scalability, and device complexity. The intentions of this review are 1) to provide a practical overview of successful FET sensor integration approaches, 2) to identify crucial challenges and factors limiting the extent of FET sensor integration, and 3) to highlight promising perspectives for future developments of FET sensor integration. We believe that our structured insights will be helpful for scientists and engineers of various profiles focusing on the design and development of FET-based chemo- and biosensor devices.

The development of micro- and nanotechnologies and the advances in materials science in the last decades have majorly impacted the architecture and performance of field effect transistors (FETs), dramatically reducing their dimensions. While the FETs are primarily designed as logic gates and switches for digital electronics, they also found important applications in analog electronics as components of low-noise and radio frequency amplifiers. The merge of nanotechnological approaches with life sciences unveiled new possibilities for using transistors in the analog domain, for example, in biosensorics to detect the concentrations of biological and chemical species with ultrahigh sensitivity, or to monitor long-term biochemical processes. This is possible due to the sensing mechanism relying on the electrostatic gating of the semiconducting FET channels by surface charges in their vicinity. Attachment of different (bio)chemical species near the channel surface causes changes in the amount and distribution of surface charges, shifting the surface potential and modulating electric current flow through the FET channel. Therefore, diverse species such as ions, molecules, or even cells, staying in close proximity to the surface of the semiconducting channel of the FET can be detected. As a consequence of the sensing mechanism, the highest sensitivity is achieved in the case of nanoscopic FETs, where surface charges can efficiently gate the entire volume of the semiconductor channels. Thus, multiple nanoscaled FET devices have been positioned as the so-called “label-free”^1^ biosensors, detecting different (bio)molecules such as neurotransmitters,^2−4^ hormones,^5^ small-molecule drugs,^6^ glucose,^7,8^ nucleic acids,^9^ and proteins,^10−13^ down to subfemtomolar concentrations.

Despite numerous successful demonstrations of label-free FET-based chemo- and biosensors, these devices still face significant challenges that create a gap between functional laboratory prototypes and commercial sensing devices that can be manufactured at scale. At the fabrication level, these devices still suffer from device-to-device variations and insufficient reproducibility in the performance of functional sensing layers. The high sensitivity of FET sensors and their required operation regimes introduce additional challenges related to hardware interfacing, including conditioning and processing electronics, reliable multiplexing and readout, and integration into fully functional standalone electronic devices.

While the functionalization strategies, sensitivity, and multiplexing ability of the FET bio- and chemosensors have been discussed in multiple reviews and original articles before,^2,14−26^ the integration of bioFETs was not systematically summarized and analyzed. A potential reason for this is the relative complexity of bridging the gap between fundamental research and the commercialization of fully functional biosensing FET devices.

Therefore, this review article analyzes diverse approaches to the integration of biological and chemical FET sensing devices into more complex systems and circuits, including microfluidics for the delivery of liquid samples. Such devices must operate intuitively and be adjusted for various application scenarios to quickly and reliably inform the user about the measurement outcomes, commonly in the point of care (PoC) and diverse “in the field” analysis settings. Ultimately, creating sufficiently sensitive, specific, accurate, rapid, robust, portable, and miniature tools that integrate FET chemo- and biosensors as key measurement interfaces is imperative. The integration depends on the key features of the individual FET sensor unit, such as the material properties of the sensing element and the underlying substrate, the FET fabrication process and its scalability, as well as on many other factors including intended device architecture, measurement media, and the surrounding environment in which the device should operate. The selection of materials for the substrate and sensing elements defines the possible FET sensor fabrication methodologies. In turn, fabrication and integration strategies for rigid and planar substrate/sensing element systems are typically directly inherited or adapted from microelectronics. However, application-specific and nonstandard fabrication protocols need to be established for flexible or rigid nonplanar substrates. These protocols are frequently *not scalable and compatible* with high integration levels. Developing adequate approaches for the fabrication and integration of FET sensors intended for wearable or implantable sensing devices is particularly challenging. Wearable devices typically require flexible and/or stretchable sensor components that provide mechanical compatibility with the measurement site, as well as a stable and reliable response in a dynamic and often physicochemically complex environment. These requirements are commonly extended even further for implantable devices which are required to operate in a demanding (bio)chemical environment rich in interfering compounds. Therefore, innovative and application-tailored approaches are continuously being developed to tackle the integration of FET sensors in wearable and implantable systems.^5,13,27−31^ One of the approaches to advance the integration levels in FET sensors is the change of inherent device architecture. Great success in simplifying the integration has been achieved using the extended-gate (EG) FET format,^32^ which allows the creation of arrays of electrodes instead of FET arrays. These extended gate electrodes can be placed in a physically separated chip and electrically coupled with a single FET transducer for cost-effective operation in a multiplexed format that easily reaches small- to medium-scale integration.^25^ For a more detailed overview of various EG-FET chemo- and biosensor aspects, the reader is referred to our previous review covering this topic in considerable detail.^24^ This review mainly focuses on classical FET chemo- and biosensor configurations, except for a few notable exceptions.

With adequate levels of integration, many features of measurement systems relying on FET sensors can be enhanced, such as versatility, accuracy, response time, automation, and user-friendliness. These developments could unlock the potential for routine use in highly demanding applications that require stable operation, including comprehensive multimarker detection for personalized diagnostics and screening, advanced therapy monitoring, theranostics, and analysis of various unprocessed fluid samples with complex chemical composition, by building upon the promising sensing concepts.^33−36^

Integration of FET chemo- and biosensors is multifaceted, and different formats of integration can be considered, such as 1) electronic integration (incorporation into a supporting standalone electronic device and formation of FET sensor arrays), 2) fluidic integration, and 3) integration into multisensor systems or devices with specific application requirements (e.g., wearable or implantable systems). Each integration type brings about a set of specific advantages, while simultaneously increasing the overall complexity of sensing device design and construction. Therefore, the desired integration formats and levels should be optimized and tailored based on the specific application demands.

## Electronic Integration of FET Sensors

Electronic integration is the most important format that defines the ability to create a functional standalone sensing device and its potential applications. Generally, electronic integration should enable a robust measurement chain allowing accurate, reproducible, and timely data acquisition, as well as seamless data transfer. The electrical signal generated at the FET-based sensing element should be transduced, conditioned, processed, and finally, the collected measurement data should be stored and/or transferred for further analysis. More specifically, FET biosensors are expected to be positioned as important starting points of data collection at the PoC within the Internet of Medical Things (IoMT) framework, one of the key components in digital health ecosystems of the future.^37,38^

**Figure 1:**
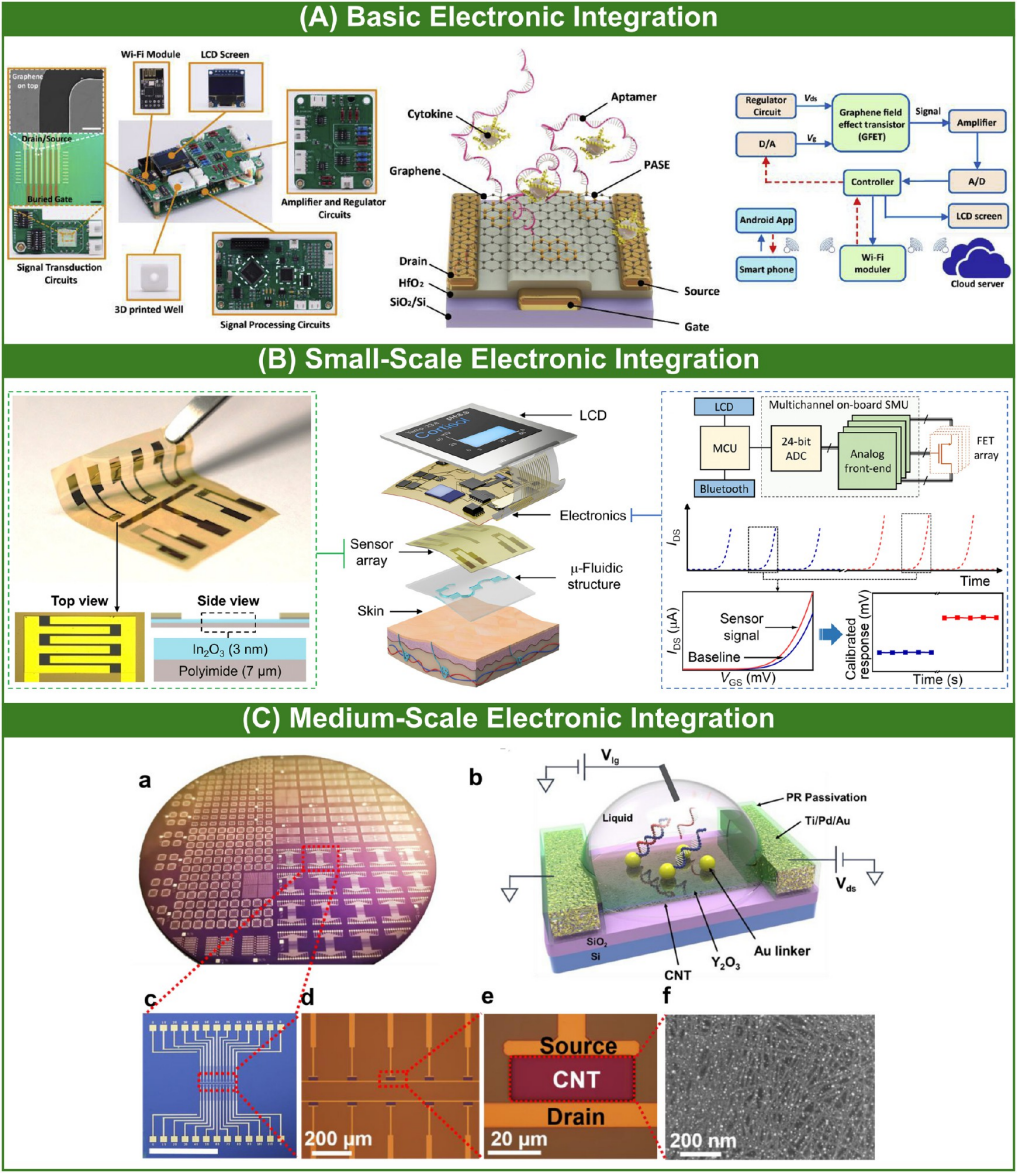
Examples of different electronic integration levels of FET chemo- and biosensors (from basic to medium scale). (A) Aptamer-modified graphene FET biosensor employing buried-gate geometry with a HfO_2_ dielectric layer to detect cytokines in saliva. Reproduced with permission from ref (53). Copyright 2019 Elsevier. (B) Array of surface-modified nanometer-thin-film In_2_O_3_ FETs for the measurement of pH and cortisol in sweat and saliva. Reproduced from ref (5). Available under CC BY 4.0 license. Copyright 2022 Wang et al. (C) Wafer-scale fabrication of highly uniform and reliable CNT FET biosensors with floating gate structure for the detection of Patau syndrome DNA sequences and microvesicles from HepG2 cells. Reproduced from ref (66). Copyright 2020 American Chemical Society.

The initial development of highly sensitive FET sensors relied on Si nanostructures, mainly focusing on Si nanowires (SiNWs) due to their high surface-to-volume ratio, small footprint, and strong compatibility with traditional complementary metal-oxide semiconductor (CMOS) processing.^39,40^ SiNW-based FET sensors have been designed to detect diverse target analytes, such as small-sized (bio)molecules,^41−43^ nucleic acids,^44−46^ and proteins.^47−49^ For example, Ma et al.^49^ implemented a rapid (2–5 min) and ultrasensitive SiNW FET biosensor to detect *Mycobacterium tuberculosis* Ag85B protein in diluted sputum. The sensor could reach a limit of detection (LOD) down to 0.33 aM and operate in an extensive dynamic range (up to 5 orders of magnitude for diluted sputum). Conveniently, an array of 160 SiNWs was arranged to form a sensing element that was efficiently packaged on a portable 13 mm × 13 mm chip, and ready for further electronic interfacing. Beyond typically rigid Si-based FET sensors, Ditte et al.^50^ have shown a realization of the organic FET biosensor based on an intrinsically stretchable (up to 90% without cracking) semiconducting triblock copolymer that is reliably functionalized by a physical adsorption method to detect SARS-CoV-2 related analytes. The semiconducting polymer layer is suitable for roll-to-roll printing, opening opportunities to create cost-effective wearable devices. Farahmandpour et al. have demonstrated ultrasensitive FET-based glucose biosensors fabricated on flexible polyethylene terephthalate (PET) substrates using hybrid metal oxide (ZnO/CuO) nanostructured hollow spheres with immobilized glucose oxidase^51^ and NiWO_4_ microcrystals^52^ as sensitive channel materials. Besides the remarkable capability for glucose detection in real blood serum samples with the LOD down to the ∼ nM range,^52^ the authors also demonstrate efficient integration into a portable device with wireless data transmission ability that enables the use embedded into the IoMT framework.^51^ Hao et al.^53^ (Figure 1A) designed an aptamer-modified graphene FET biosensor employing buried-gate geometry with an HfO_2_ dielectric layer to detect cytokines in saliva within minutes with 12 pM LOD for IL-6 as a model analyte. The authors realized a completely portable and modular nanosensing system that enables wireless transmission of data to the smartphone or cloud server and a Universal Serial Bus (USB) connection to the computer.^53^ Wang et al.^54^ have reported an ultrasensitive detection of diverse analytes in biofluids using an electromechanical principle relying on a complex molecular system (which comprises a tailored deoxyribonucleic acid (DNA)-based cantilever structure coupled to an aptamer probe) immobilized on a graphene FET. Using this approach they created a battery-powered prototype testing system for SARS-CoV-2 nucleic acids with a disposable sensing interface and reusable supporting electronics enabling multiple types of connectivity between the computer or smartphone and the sensing device (via USB, Bluetooth, or WiFi).^54^

The initial demonstrations of individual FET sensors incorporated into standalone electronic devices that provide measurement and data transfer functionalities can outline some promising prototype design approaches. However, such design strategies are often not compatible with the integration into FET sensor arrays and corresponding electronic interfacing. Implementation of FET sensor arrays requires reliable fabrication with higher FET device density and smaller footprint coupled with more complex interfacing electronics for multiplexing and readout.

From a practical point of view, electronic sensing devices of higher complexity involving the formation of FET sensor arrays bring about numerous important advantages. These advantages include 1) higher testing throughput coupled with smaller analyte sample volume, 2) reduced footprint and form factor of advanced devices, 3) simultaneous measurements of multiple analytes, 4) introduction of simultaneous baseline or reference measurements to correct for interference effects, 5) improved reliability through defined repeatability, cross-validation, and redundancy, 6) robust statistical analysis (particularly for large scale arrays), and 7) capability to generate large amounts of data for artificial intelligence-assisted processing and analysis. Designation of the integration level in the case of FET sensor arrays can be borrowed and adapted from metal-oxide-semiconductor field-effect transistor (MOSFET) integration in microelectronics, by replacing the number of transistors in integrated circuit chips with the number of individual FET sensor units comprising the sensing interface. In this case, the integration levels can be classified as follows:^55^

- Small-Scale Integration (SSI, <10^2^ units)
- Medium-Scale Integration (MSI, 10^2^–10^3^ units)
- Large-Scale Integration (LSI, 10^3^–10^4^ units)
- Very Large-Scale Integration (VLSI, 10^4^–10^5^ units)
- Ultra Large-Scale Integration (ULSI, 10^5^–10^7^ units)
- Super Large-Scale Integration (SLSI, >10^7^ units)

Measurements on FET arrays are typically carried out using electrical multiplexing. Among many electrical multiplexing approaches (spatial, time-division, frequency-division, barcode, and particle-based), spatial multiplexing dominates due to its simplicity and versatility.^56,57^ Facile and well-established electronic readouts can be used, while sensing units can be arranged in various architectures, allowing for rapid data collection from a complete sensor array. Spatial multiplexing for lower degrees of integration is typically realized as the front end of the analog-to-digital converter (ADC)^58^ or as a separate electronic module.^25^ In biosensing devices featuring large-scale integration, more sophisticated addressing of individual sensing units is necessary, and it is often realized using readout strategies established for CMOS-compatible arrays.^26,59^ The main disadvantages of spatial multiplexing are the sequential readout limiting acquisition rate and the challenges of creating identical measurement interfaces for all individual sensing units. Depending on the level of integration, measurement interface variations can be resolved using a robust design of multiplexing electronics,^25^ advanced CMOS fabrication processes,^60^ or dedicated calibration strategies.^61^ The peak acquisition rate reduces proportionally to the number of sensing units and can reach minutes for ULSI FET biosensors.^26,60^ In addition, there is also a less common approach of partially or fully parallel measurements. Parallel measurements are often impractical due to highly demanding acquisition hardware and required processing power, both being limited in conventional portable electronics. However, if the high acquisition rate and measurement timing are critical, some degree of parallel processing may become necessary. The inherent relatively low rate of (bio)chemical processes in biosensors does not present a significant challenge for traditional multiplexed measurement targeting stable states of biosensor response. Conversely, timely data acquisition and processing may pose a challenge in specific cases of demanding application scenarios, e.g., involving continuous monitoring of rapidly changing analyte concentration,^4,10,62^ advanced analysis of drug binding kinetics,^6^ or monitoring of dynamic physical parameters^3,5,7^ in flexible or wearable devices (temperature, pressure, strain, humidity, etc.) to correct for their effects on the FET chemo- and biosensor response.

### Small-Scale Electronic Integration of FET Sensors (<10^2^ units)

Most device implementations using FET chemo- and biosensor arrays still fall into the small-scale integration category. Such devices are easier to realize in a format comparable with traditional laboratory-scale assays that are considered gold standards in the sensing of chemical and biological analytes, such as enzyme-linked immunosorbent assays (ELISAs) and assays based on polymerase chain reaction (PCR). The research focus has shifted to demonstrate the SSI and multiplexing of FET biosensors based on emerging low-dimensional materials, such as graphene, carbon nanotubes (CNTs), MoS_2_, In_2_O_3_, and indium gallium zinc oxide (IGZO), as well as the organic FETs.

Xu et al.^6^ have demonstrated a single-crystal graphene-based FET sensor as a tool to assess the interaction of the low molecular weight drug imatinib that is used to treat chronic myeloid leukemia with its target protein Abl1 in various in vitro environments (including human serum) that may affect the drug binding affinity and kinetics. The sensor proved highly sensitive with the LOD for imatinib of 15.5 fM and a broad dynamic range with linear response versus the logarithm of imatinib concentration (0.1 pM–10 μM).^6^ Graphene multitransistor arrays modified with selective DNA aptamer were also used for ultrasensitive dopamine detection with the detection limit down to 1 aM in artificial cerebrospinal fluid and covering a remarkable concentration range spanning 10 orders of magnitude.^4^ The array of 20 electrolyte-gated graphene FETs was integrated on a single chip, followed by gluing and wire-bonding to interface with the custom printed circuit board (PCB) designed for simultaneous measurement of independent sensing units providing robust statistics.^4^ Aptamer-functionalized solution-gated graphene-based FETs were utilized in a similar setup to achieve aM detection of the core protein of the hepatitis C virus in human blood plasma.^63^ Kumar et al.^64^ demonstrated a chip integrating 4 liquid-gated graphene FETs with a common source electrode that was implemented for simultaneous measurement, achieving highly sensitive detection of SARS-CoV-2 spike (LOD of 88 zM) and H3N2 Influenza (LOD of 227 zM) surface proteins with a rapid response time of ∼ 10 s in 0.01 × phosphate-buffered saline (PBS). The chip integrates 2 graphene FETs functionalized with corresponding antibodies and 2 control FETs (with bare and chemically passivated graphene) making it suitable for differential diagnosis.^64^

He et al.^8^ implemented an aptamer-functionalized floating-gate CNT-based FET with a deposited Y_2_O_3_ layer, serving as an ultrasensitive glucose sensor (LOD of 0.5 fM) with a broad linear response range (9 orders of magnitude) and low power consumption (<100 pW). The authors demonstrated an array of 22 sensors with good reproducibility, where glucose-binding aptamers are attached to the FET channel using assembled gold nanoparticle-based linkers on the Y_2_O_3_ layer, and sensitivity can be enhanced by tuning the gate-to-source voltage.^8^ The proposed sensing paradigm is attractive for detecting small-sized biomarkers carrying low or neutral charge in aqueous solutions with physiological ionic strength. Similar FET biosensor design and aptamer linking strategies were used to detect Alzheimer’s disease^62^ and COVID-19^9^ biomarkers. The β-amyloid blood biomarkers (Aβ_40_ and *Aβ*_42_) could be reliably detected (variation smaller than 10% for 10 devices) already at sub-fM levels in undiluted human blood serum with fast response time (∼min) and relatively broad dynamic range (4 orders of magnitude).^62^ SARS-CoV-2 surface antigen and ribonucleic acid (RNA) were detected simultaneously and directly from nasopharyngeal swab samples within 1 min using an FET array fully integrated into the electronic chip and further embedded into the portable point-of-care testing device.^9^ The FET biosensor architecture coupled with the readout relying on precise drain current amplification and low-pass filtering enabled RNA detection down to a single virus level without any sample amplification and processing.^9^ Biofunctionalization and environmental stability of CNT FET sensors can be improved by constructing a high-κ dielectric bilayer comprising Y_2_O_3_ and HfO_2_ as gate insulators. A pH sensing array with this architecture comprising 17 FET sensors showcased the capability for continuous pH monitoring in a wide pH range (1.34–12.68) exhibiting low hysteresis, good reproducibility, and super-Nernstian sensitivity of 67.62 mV/pH.^65^ The same FET architecture was employed to construct an array of 8 FET biosensors for ultrasensitive and real-time detection of cardiac troponin I, a biomarker of acute myocardial infarction.^10^ To achieve specific and ultrasensitive detection (LOD of 0.33 fg/mL) even in blood plasma samples, the FET channel was decorated with Au nanoparticle linkers and then further modified with the clustered regularly interspaced short palindromic repeats (CRISPR)/Cas12a system involving the G-triplex reporter.^10^ The biosensing FET array interface was integrated into a testing chip that is embedded within a standalone portable electronic readout device, providing the response within 2 min for clinical samples.^10^

Yang et al.^11^ have demonstrated an MoS_2_ nanosheet-based FET sensor array comprising four sensing windows (each with multiple FET sensing units) for the ultrasensitive detection of two bladder cancer biomarkers (nuclear matrix protein 22 and cytokeratin 8) in processed and diluted human urine. The created biosensor array features a broad linear range (from 10^–6^ to 10^–1^ pg/mL) and remarkable detection limits reaching down to ∼ 10 zM range.^11^ Wang et al.^5^ (Figure 1B) have reported a biosensor array of nanometer-thin-film In_2_O_3_ FETs that can be used for the measurement of pH within the physiological range when functionalized with (3-aminopropyl) triethoxysilane and specific cortisol detection with appropriate aptamer-based modification in sweat and saliva. The sensing system is integrated into an autonomous wearable point-of-care device that comprises an onboard multichannel high-resolution source measure unit implementation, liquid crystal display, and hardware for Bluetooth communication. The device enables real-time monitoring of cortisol concentration in a wide range (1 pM–1 μM), pH, and temperature from an integrated sensor.

Zhang et al.^67^ have demonstrated a sensing array of 8 IGZO-based FET biosensors enabling multiplexed specific detection and discrimination of SARS-CoV-2 variants rapidly (<15 min) and with high sensitivity (LOD: 0.03 copies μL^–1^). To endow the sensing system with such capabilities, the FET sensors were functionalized using paperclip-shaped nucleic acid probes allowing for highly precise identification of RNA mutations at the single nucleotide resolution level.

Macchia et al.^68^ have reported a small-scale array of large-area electrolyte-gated organic FETs relying on inkjet-printed semiconducting channels composed of poly(3-hexylthiophene) and patterned on poly(ethylene 2,6-naphthalate) substrates with gold contacts that features very good reproducibility (deviation below 3%). The system showed LOD down to the ∼ 10 zM range in PBS and whole human blood serum for biomarkers of mucinous pancreatic cysts such as KRAS oligonucleotide and oncoprotein Mucin1. The designed three-dimensional (3D) FET biosensor architecture can be also made compatible with the standard ELISA plates as shown by Sarcina et al.^69^ in another study.

#### Critical Remarks

The exemplary studies showcased above illustrate that many FET sensing devices featuring SSI are capable of detecting analytes even in raw or slightly processed complex samples. Despite their demonstrated remarkable detection capabilities, FET sensing devices with SSI levels need additional improvements. It must be noted that the fabrication of FET sensor arrays based on innovative low-dimensional and organic materials still suffers from significant device-to-device and batch-to-batch performance variations. Although the small-scale fabrication of FET sensor arrays is demonstrated using various techniques, ∼ 10% of device-to-device sensing performance variation remains typical even for adjacent sensors produced within the same batch. Thus, further improvements and simplifications of fabrication and surface modification methods are required to create robust production processes. In addition, issues related to device variations and interferences arising from complex analyte sample composition could be partially alleviated through different artificial intelligence algorithms for the adjustment of sensor calibration and validation of sensing response reliability.

From a design perspective, exciting emerging trends in the systems with small-scale electronic integration are hydrogel-based gating and realizations of lab-on-PCB systems. Hydrogel sensing interfaces offer opportunities for real-time, specific, and selective detection of analytes in complex media and represent a special biointegration strategy.^7,70,71^ The hydrogel gating elements can be produced using additive manufacturing techniques (e.g., microfluidic dispensing or 3D printing coupled with photopolymerization)^70,71^ or strategies of self-assembly^7^ on the surface of the sensing element. Hydrogel gating is a scalable strategy that has so far proved to be suitable for the detection of glucose, enzymes (penicillinase and acetylcholinesterase), penicillin, and urea.^7,70,71^ Papamatthaiou et al.^72^ have demonstrated the possibility of constructing an array of 12 electrolyte-gated graphene FET biosensors for label-free detection of DNA on a commercially fabricated PCB. Fabrication of the graphene channel and immobilization of the peptide nucleic acid probes were performed using processes fully compatible with inkjet printing, thereby facilitating the rapid integration of FET biosensors directly on the PCB. An in-plane Ag/AgCl reference electrode with satisfactory performance was also integrated into the measurement system. This study showcases the feasibility of facile fabrication and seamless integration of FET sensors within Lab-on-PCB systems, thereby paving the way for sensing platforms of higher complexity. More modular multiplexed FET biosensing systems obviating the need for dedicated FET fabrication can be implemented using the EG FET paradigm.^25,73^

### Medium-Scale Electronic Integration of FET Sensors (10^2^–10^3^ units)

Compared with small-scale electronic integration realizations, which emerge mainly for novel classes of low-dimensional materials, already-established materials are typically explored for different large-scale integration levels. Therefore, MSI is not a common focus in developing FET sensing systems, except for FET array fabrication reported at a wafer scale. In the context of FET sensor array fabrication, the ability to fabricate multiple FET sensing units covering the surface area of a standard commercial silicon wafer indicates scalability and a degree of compatibility with conventional industrial workflows. Notably, this wafer-scale fabrication should not be always considered as a direct equivalent of traditional wafer-scale integration. There are a few recent notable studies showcasing the feasibility of MSI of FET sensors based on Si structures,^28,74,75^ CNTs,^66,76^ and graphene.^77^ Midahuen et al.^74^ have reported a CMOS-compatible fabrication of bioFET arrays based on Si nanostructures (nanowires, honeycomb structures, and nanoribbons). They investigated the performance of different nanostructures for pH sensing relying on ion-sensitive (IS) FETs and reached optimal performance for nanoribbons in dual-gate configuration with super-Nernstian sensitivity of 600 mV/pH. The authors also demonstrated a 10 × 10 Si nanoribbon-based bioFET array for the sensitive electrical detection of DNA hybridization achieving a threshold voltage shift of 200 mV. Lai et al.^75^ implemented a 16 × 16 array of label-free capacitive FET biosensors for the detection of pH and DNA hybridization using a CMOS-compatible fabrication process. The sensor array was wire-bonded to the dedicated readout PCB. In the reported configuration, individual sensors were activated using row and column decoders, read out with switched-capacitor circuits, and then digitized using AD conversion circuitry. Measurements of pH performed in the study reached the sensitivity of 151 fF/pH while the measurements of DNA hybridization achieved a sensitivity of 94 fF/log10[DNA] in the concentration range from 10 aM to 100 pM at a clock frequency of 2 MHz. Liang et al.^66^ (Figure 1C) reported a wafer-scale fabrication of highly uniform and reliable CNT FET biosensors with a floating gate structure employing an ultrathin high-κ dielectric layer of Y_2_O_3._ The FET biosensors were arranged as clusters of arrays with 22 sensing units across the wafer. The FET devices could be fabricated with 100% yield and very high semiconducting purity (>99.9%) of single-wall CNTs. The authors reported biosensing of specific DNA sequence characteristic for Patau syndrome and microvesicles derived from HepG2 cells with remarkable sensitivities achieving theoretical detection limits of 60 aM and 6 microvesicles/mL, respectively. Similarly to wafer-scale Si-based fabrication, large-area fabrication of CNTs could be realized on plastic substrates using cost-effective technologies. Sun et al.^76^ demonstrated the fabrication of fully printed FET sensor arrays assembled on the polyethylene naphthalate substrate modified with parylene C using gold nanoparticle and semiconducting single-wall CNT inks as key components. The obtained FET sensor arrays were suitable for MSI and exhibited a yield of 100%. The authors showed a label-free detection of negatively charged bacteria *Shewanella onedensis* MR-1 in 0.1 μL of suspension at the LOD of 10^5^ CFU/mL and with the sensitivity of 14%/dec expressed as the relative change in the current response. Soikkeli et al.^77^ demonstrated full wafer-scale integration of 512 graphene FETs with a CMOS multiplexer enabling simultaneous resistive readout for chemosensing of different NaCl concentrations. The constructed integrated device features a yield of 99.9%, good reproducibility of device characteristics, and a statistically robust response in the NaCl concentration range from 1 to 100 mM (sensitivity of 42 ± 4 mV/dec). The reported platform design can be extended to other applications based on functionalized graphene FETs, such as gas sensing and infrared imaging systems.

#### Critical Remarks

Significant advances at the MSI level can be noted in the fabrication of FET sensor arrays that rely on carbon-based low-dimensional materials, even when using cost-effective fabrication methods based on solution processing and flexible polymer substrates. We also observe the emergence of interesting readout and interfacing methods, mainly inspired by simplified or adapted concepts that are well-established for LSI-level systems. However, FET sensor systems at the MSI level still require improved and tailored readout strategies that are cost-effective and compatible with standalone device operation. The hardware interfacing and readout remain particularly challenging for medium-scale FET sensor arrays printed on flexible substrates, where traditional electronic interfacing cannot be directly applied, and mechanical effects need to be carefully compensated.

### Large-Scale Levels of Electronic Integration in FET Sensors (>10^3^ units)

**Figure 2:**
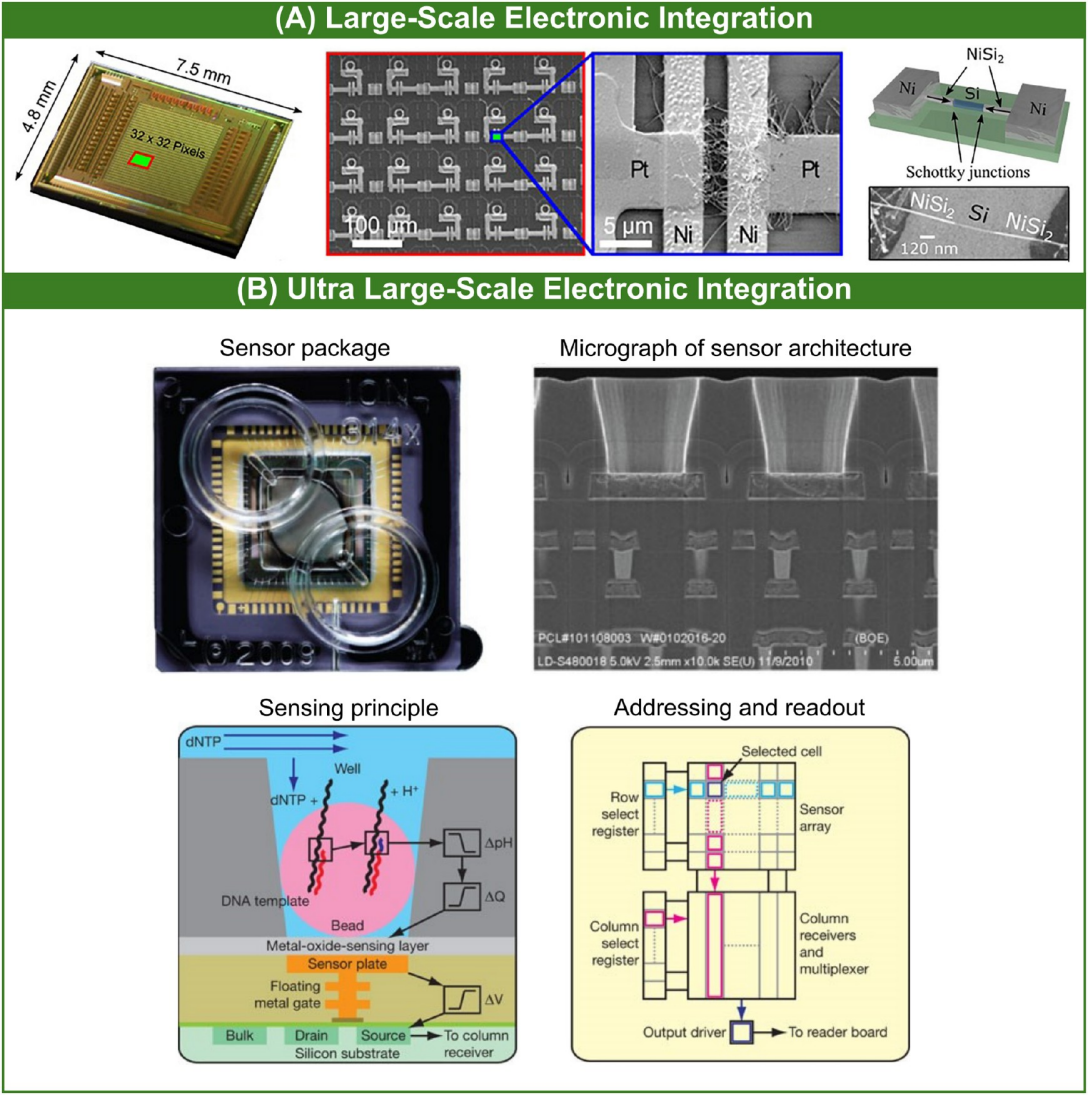
Examples of large-scale and ultra large-scale electronic integration levels of FET chemo- and biosensors. (A) Array of 32 × 32 bottom-up silicon-nanowire Schottky-junction FET sensing units for the ultrasensitive detection of dopamine. Reproduced from ref (2). Available under CC BY 4.0 license. Copyright 2022 Sessi et al. (B) Commercialized system for rapid DNA sequencing based on large-scale IS FET arrays (Ion Torrent). Reproduced from ref (59). Available under CC BY-NC-SA 3.0 license. Copyright 2011 Rothberg et al.

With the increasing level of electronic integration, the number of reported studies in the literature becomes scarcer as large-scale integration brings about various challenges regarding FET chemo- and biosensor fabrication and interfacing with adequate supporting electronics for multiplexing and readout. The high levels of integration predominantly rely on well-established Si-based FETs that are known to be compatible with traditional CMOS processing. Looking into different degrees of large-scale integration, there are a few notable studies and most of them represent a transition toward industrial and commercial developments. Sessi et al.^2^ (Figure 2A) have demonstrated the so-called hybrid integration of an array of 32 × 32 bottom-up silicon-nanowire Schottky-junction FETs as sensing units with 100 μm pitch and CMOS-compatible electronics for readout and signal amplification. The authors achieved a yield of up to 85.1% of functional FET devices that were interfaced with a customized FPGA reader enabling simultaneous recording of 32 FET transfer characteristics and row-wise scanning, leading to the scanning of a complete sensing array within a few min. Using aptamer-based functionalization and subthreshold regime measurements in diluted PBS, the detection of dopamine was showcased at an LOD in the fM range with a remarkable peak sensitivity of ∼ 1 V/fM. Bashir group and collaborators have developed two platforms comprising 1024 × 1024 arrays of FET sensors,^26,60^ both fabricated in the foundries of Taiwan Semiconductor Manufacturing Company (TSMC) using a standard CMOS process. Duarte-Guevara et al.^26^ realized the massively multiplexed array of dual-gated BioFETs with 6.5 μm pitch on a 7 × 7 mm^2^ chip area with on-chip circuits for row and column addressing. The chip was wire-bonded to a PCB with 256 pins that enabled interfacing compatibility with the PXI logic integrated circuit (IC) tester. PCB contained a conditioning trans-impedance amplifier for drain current signal amplification and conversion to voltage that connects with the readout stage for serial measurement operating with a period of ca. 0.11 ms, thereby leading to a full scan of the array within 90 s. To characterize the platform and demonstrate its functionality, the authors performed pH measurements relying on a HfO_2_ layer in the range 4–10. Results demonstrated good stability of the response, resolution of 0.25 pH units, and spatially variable sensitivity of 45.8 ± 5.4 mV/pH (single-gate mode) that can be amplified up to 84 mV/pH when using dual-gate operation. They also showcased the effective reduction of response variability using the techniques of redundancy and differential referencing. Ganguli et al.^60^ reported the ultralarge-scale array of PNA-probe functionalized IS-FETs for the detection of microRNA (Let-7b) based on the hybridization reaction. By monitoring the modulation of drain current during hybridization using a similar acquisition approach as in the previous study,^26^ the authors reached the LOD down to 1 nM and showcased the excellent robustness of the system. The recorded drain current for over a million integrated IS-FET devices showed a standard deviation <13% of the mean, indicating a very uniform sensing response. The system also proved as highly reliable and resistant to noise contributions, showing a significantly stronger response for specific detection of microRNA compared to noncomplementary RNA and control (*p*-value lower than 0.0001). An important commercialized IS-FET-based system for DNA sequencing^59^ (Ion Torrent^78^) (Figure 2B) relying on high levels of electronic integration (ULSI and SLSI) using CMOS-compatible manufacturing will be presented in more detail later in the text.

#### Critical Remarks

Compatibility of Si-based FET sensors with CMOS processing has opened many interesting opportunities to form large-scale arrays of chemo- and biosensors and simultaneously enabled the use of well-known strategies for effective electronic interfacing and readout. However, some limitations to integration still exist in these systems. For example, in the so-called hybrid integration with SiNW-based FET sensors, the diversity of SiNWs leads to significant variations in device-to-device performance and discrepancies in contact resistance reduce the signal-to-noise ratio at the output. For large-scale FET sensor systems fabricated using conventional CMOS processing, we can still observe notable variations in sensing performance (∼10%) and the demonstrated applications remain limited to simple scenarios such as pH sensing and detection of nucleic acid hybridization reactions. Although the electronic interfacing and readout are highly efficient, the demonstrated solutions are typically bulky and not optimized for portable standalone devices. Therefore, FET sensing systems with high integration levels also require improvements in terms of fabrication and miniaturization of supporting electronics, as well as additional development efforts focusing on relevant applications in the biomedical field, particularly clinical diagnostics and screening.

## (Micro-) Fluidic Integration of FET Sensors

As FET-based chemo- and biosensors are typically exposed to different liquids during functionalization, processing, and measurements, fluidic integration is an additional beneficial integration format. It is well-known that fluid flow can significantly and systemically affect the response of electrochemical sensors,^79^ and even be intentionally tailored to improve the features of biosensor response.^80^ Modern flow control systems allow automated fluid delivery, accurate dosing of liquids, precise tuning of flow rates, and controllable flow multiplexing, thereby effectively contributing to the more reproducible behavior of biosensing interfaces and minimal consumption of analytes or reagents. Therefore, fluidic integration brings about multiple advantages: 1) automation of many processes, such as sample pretreatment and delivery, surface modification or functionalization, washing and regeneration, and measurement conditioning; 2) capability to monitor dynamic processes and measure under diverse fluid flow conditions; 3) possibility to optimize stability and signal-to-noise ratio for electrochemical sensing in liquids; 4) precise flow control and multiplexing enabling localized processing and measurements unavailable in traditional reservoir systems.

**Figure 3:**
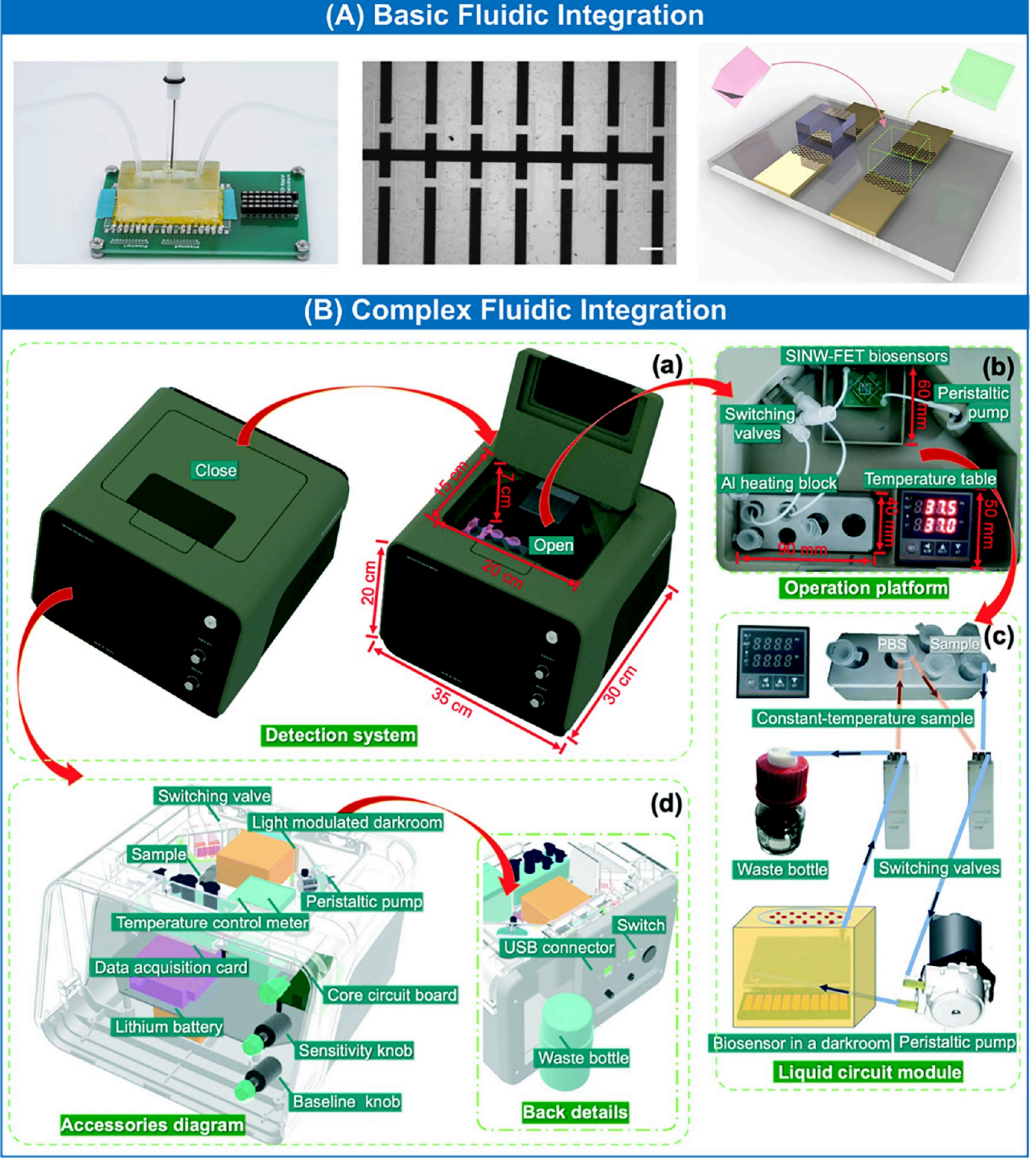
Examples of fluidic integration formats for FET chemo- and biosensors and their relation to electronic integration. (A) 3D printed chamber accommodating fluidic inlet and outlet for liquid delivery together with the placement of the external Ag/AgCl reference electrode; the chamber is aligned with graphene FET sensors and allows the assembly of encoded hydrogel stamps as biorecognition modules. Scale bar: 200 μm. Reproduced from ref (71). Copyright 2019 American Chemical Society. (B) Complex liquid circuit module fluidically integrated with the SiNW FET immunosensor for the detection of *Mycobacterium tuberculosis*. Reproduced with permission from ref (87). Copyright 2022 Royal Society of Chemistry.

In its simplest form, fluidic integration includes a single microfluidic channel spatially aligned with FET sensors, enabling liquid delivery for dosing or dynamic testing purposes. Panahi and Ghafar-Zadeh used a mm-scale 3D printed mold to create a polydimethylsiloxane (PDMS) channel for liquid delivery to the Open-Gate Junction FET pH sensor.^81^ Wang et al.^65^ employed a simple single-channel PDMS module to test the static response stability after electrolyte injection and dynamic response to real-time pH changes for the CNT FET-based pH sensor. Xu et al.^6^ reported the use of a single channel that is CNC-machined in poly(methyl methacrylate) (PMMA) and bonded using ultraviolet (UV) light-curable adhesive for liquid injection and removal from the graphene FET sensors designed for imatinib detection. This channel was used for syringe pump-controlled liquid supply and exchange during functionalization and measurement. Dai et al.^71^ (Figure 3A) implemented a simple resin-based 3D printed chamber accommodating fluidic inlet and outlet for liquid delivery together with the placement of the external Ag/AgCl reference electrode for electrolyte gating. The chamber was aligned with graphene FET sensors to allow for the assembly of encoded hydrogel stamps on the sensing surface as biorecognition modules. Song et al.^12^ realized a 2 × 12 array of rolled-up InN microtube-based FET sensors integrated with two separate PDMS microfluidic channels for simplified fluid handling, multiplexed detection, and hosting of common external Ag/AgCl reference electrodes utilized for gating that are tightly fitted into the outlets. Interestingly, the rolled-up microtubes in this configuration also act as microfluidic channels for liquid transport. The authors achieved the LOD of 2.5 pM for HIV g41 antibodies spiked in human serum. Kim et al.^82^ employed a dual-channel PDMS-based microfluidic device to improve and automate the detection of Gram-positive and -negative bacteria using a graphene micropattern FET. The authors demonstrated the capability for selective and reproducible real-time detection under optimized dynamic conditions of continuous fluid flow that was used to perform sample injection or exchange, washing steps, and measurement conditioning enabling the minimization of noise and contaminant reactions. Wang et al.^83^ showcased the split-float-gate architecture of the graphene FET integrated with a simple microfluidic system enabling isolated manipulation of solutions required for multiplexed biochemical functionalization through a network of microfluidic channels. As a proof of principle, the authors demonstrated multiplexed biosensing of three liver cancer biomarkers, carcinoembryonic antigen, α-fetoprotein, and parathyroid hormone with LODs in the ∼ 10 nM range. Similarly, a simple module comprising a network of independent microfluidic channels can be utilized as a tool for biofluid collection and routing for further analysis purposes. Wang et al.^5^ developed a simple skin-adherable tape-based thin-film microfluidic structure for sweat sampling that was aligned and integrated with thin-film In_2_O_3_ FET biosensors for cortisol detection fabricated on a flexible polyimide substrate. Microfluidic channel structures were created by laser patterning of the 170-μm thick double-sided tape, which was aligned and bonded with the patterned 100-μm thick PET film containing laser-cut holes that act as ejection pathways for analyzed sweat. Such a microfluidic module enabled efficient collection and routing of sweat samples that were harvested passively or actively via iontophoretic stimulation. For highly sensitive detection, a more comprehensive fluidic system can be interfaced with the FET sensor. Liu et al.^84^ reported a nanowell FET, a special architecture comprising a 25 nm-sized well situated in the center of a 35–40 nm wide Si FinFET. When electrolytically gated, this structure exhibits a remarkable subthreshold swing of 66 mV/dec and characteristics free of hysteresis, allowing it to reach the signal of up to 40 mV when detecting 20 base-long single-stranded DNA. To perform real-time and end-point measurements using the FET biosensor, the authors integrated a fluidic system featuring pressure-driven flow control of samples and reagents with 10-channel switching, degassing module, polytetrafluoroethylene flow cell with a gasket mounted on the FET sensor chip, flow-through Ag/AgCl reference electrode module for electrolyte gating, flow sensor, and waste collection. Electrical measurements were carried out using a semiautomated probe station.

Fluidic integration can also play an important role in renewing and recycling the FET-based sensing interfaces through automated fluid transport for washing steps and analyte delivery. This aspect is yet to be explored in detail with the emergence of an increasing number of reusable FET sensors. However, there are a few studies showcasing the potential renewal and recycling strategies for biosensing interfaces that could be incorporated into fluidic systems. Lei et al.^85^ demonstrated the monitoring of released Ca^2+^ in real-time using a reduced graphene oxide nanosheet FET biosensor that can be renewed by the photocatalytic self-cleaning effect of TiO_2_ nanoparticles. The protocol that achieved 2 cycles of effective regeneration was started by immersing the chip in pure water and subjecting it to UV irradiation for 1 h. To finalize the protocol, the chip was rinsed with ethanol, then three times with pure water, and finally dried with N_2_. Hence, the automatic regeneration protocol could be implemented by combining the fluidic system and mounted UV light source. Temporary functionalization for biosensing can be also attained using bioreceptor-modified magnetic particles. The concept relying on trapping and detrapping of functionalized magnetic particles by magnetic field was reported by Lee et al.,^86^ and required the application of external magnetic fields (450 mT) perpendicularly to the sensing surface for several min to attach and remove the particles. The sensing interface required intense washing after attachment (8 times using PBS) as well as after removal (ethanol and deionized water washing steps). The sensing chip could be reused for up to 8 repeated measurements. In this case, automatic regeneration protocol would require fluidic integration coupled with controlled application of magnetic fields. Wang et al.^13^ showed highly effective regeneration of the biosensing interface (up to 80 cycles) when using an FET sensor based on the graphene-Nafion composite film that can be fabricated on rigid and thin PET substrates. After each detection cycle, the Nafion-based film could be dissolved and removed by simply immersing the sensor in an ethanol solution. The sensor regeneration is then completed by repeating the drop-casting of the Nafion solution and the functionalization process. Remarkably, the achieved relative output signal variation for the equivalent concentration of the analyte after up to 80 cycles of regeneration was lower than 8.3%. For the proposed FET sensor realization, even a fully automated regeneration process based on the integrated fluidic system could be implemented in a relatively straightforward manner.

### Critical Remarks

Fluidic integration of FET sensors is still in its early stages of development. Nevertheless, some interesting demonstrations showcasing the advantages of such an integration strategy have been already reported in the literature. Although simple forms of fluidic integration are relatively easy to implement, more intricate realizations may require the design of tailored microfluidic networks, specific fabrication techniques, and dedicated pumping systems for fluid manipulation. Therefore, the integration of fluidics with FET sensors can quickly increase the complexity of device implementation and limit the opportunities to realize portable standalone sensing devices. Prospectively, fluidic integration can significantly reduce the amount of waste generated in chemo- and biosensing procedures by minimizing reagent consumption and enabling efficient regeneration or recycling of sensing interfaces. The true potential of fluidic integration is yet to be investigated to form a new generation of highly automated, accurate, and cost-effective FET-based sensing devices.

## Synergy between Electronic and Fluidic Integration of FET Sensors

In demanding applications requiring high sensitivity, accuracy, and reproducibility, it is particularly beneficial to make a strong synergy between electronic and fluidic integration. The reliability and robustness of the FET sensor performance in such cases largely depend on the compatibility and optimized interactions between electronic and fluidic systems. In particular, adequate synchronization of fluidic and electrical multiplexing is essential for stable and accurate chemo- or biosensor operation. The synergy of fluidic and electronic integration can be achieved by design, using advanced interfacing of traditional multiplexing electronics with the driving and control electronics that regulate the properties and distribution of fluid flow. With the improvement in micropumping technologies, it is now also possible to miniaturize bulky systems for fluid delivery and flow multiplexing. Hence, the synergy of electrical and microfluidic integrations unlocks the opportunities to create fully autonomous and cost-effective biosensing devices.^88^

Xie et al.^87^ (Figure 3B) demonstrated an automated, modular, and portable (35 cm × 30 cm × 20 cm) nanobiosensing device relying on SiNW FET suitable for mass fabrication. The device comprises several modules driven by a common microcontroller: a liquid circuit module, a light modulation module, a constant PID-based temperature control module, a signal acquisition and amplification module, and a status and result display module. A unique feature of this platform is the capability to use light modulation and temperature control to alleviate interference effects, assess sensor performance, and carry out sensor calibration, all of which allow suitable correction of the sensor response. The liquid circuit module contained a peristaltic pump, switching valves, a temperature-controlled sample holder, a PMMA-based microfluidic chip with an integrated SiNW FET biosensor, and a waste container. Evaluation of FET sensor performance was performed using a light modulation module at a fixed temperature before the measurements. Supporting electronics contained DC biasing circuits for the FET, filtering and amplification stages for signal conditioning, and a commercial data acquisition card. Using this highly integrated system, the authors showcased the immunosensing of *Mycobacterium tuberculosis* with a detection limit of 1.0 fg/mL and the possibility of analyzing molecular interactions on the example of the binding-dissociation of antibody–protein pairs.

Zhang et al.^89^ reported a graphene FET-based sensing device relying on electrolyte gating in reflectometry mode at ultrahigh frequencies (UHFs, ca. 2 GHz) to cancel out the ionic screening and emphasize the changes in dielectric properties during biodetection. To implement the UHF-compatible measurement setup, the authors enclosed a μm-sized graphene ribbon with a 1-μm thick microfluidic channel made of SU-8 and inserted a commercial Red Rod reference electrode for electrolyte gating. This sensing construct was then wire bonded to a dedicated PCB and a PMMA flow cell was mounted on top of the chip to form a radio frequency (RF) box. This RF box was further interfaced with a custom-built setup for UHF measurements comprising the two-channel lock-in amplifier and front-end signal conditioning electronics. The designed setup enabled the dual acquisition of the UHF interferometry and graphene resistance-related signals, representing UHF dielectric-modulated and traditional field-effect sensing, respectively. To explore the opportunities brought about by dielectric-modulated detection, the authors investigated different model systems including biotin–streptavidin binding, formation of the heterodimeric coiled-coil complex, polyelectrolyte multilayers assembled on top of graphene, and cardiomyocytes cultured on the entire graphene ribbon (5000 cells/mm^2^). Their results suggest that dielectric-modulated detection provides the capability to probe biochemical interactions far beyond the Debye length even in concentrated salt solution (e.g., up to 4 nm in 1 M KCl) and deeply into cell bodies to detect changes in contractility.

**Figure 4:**
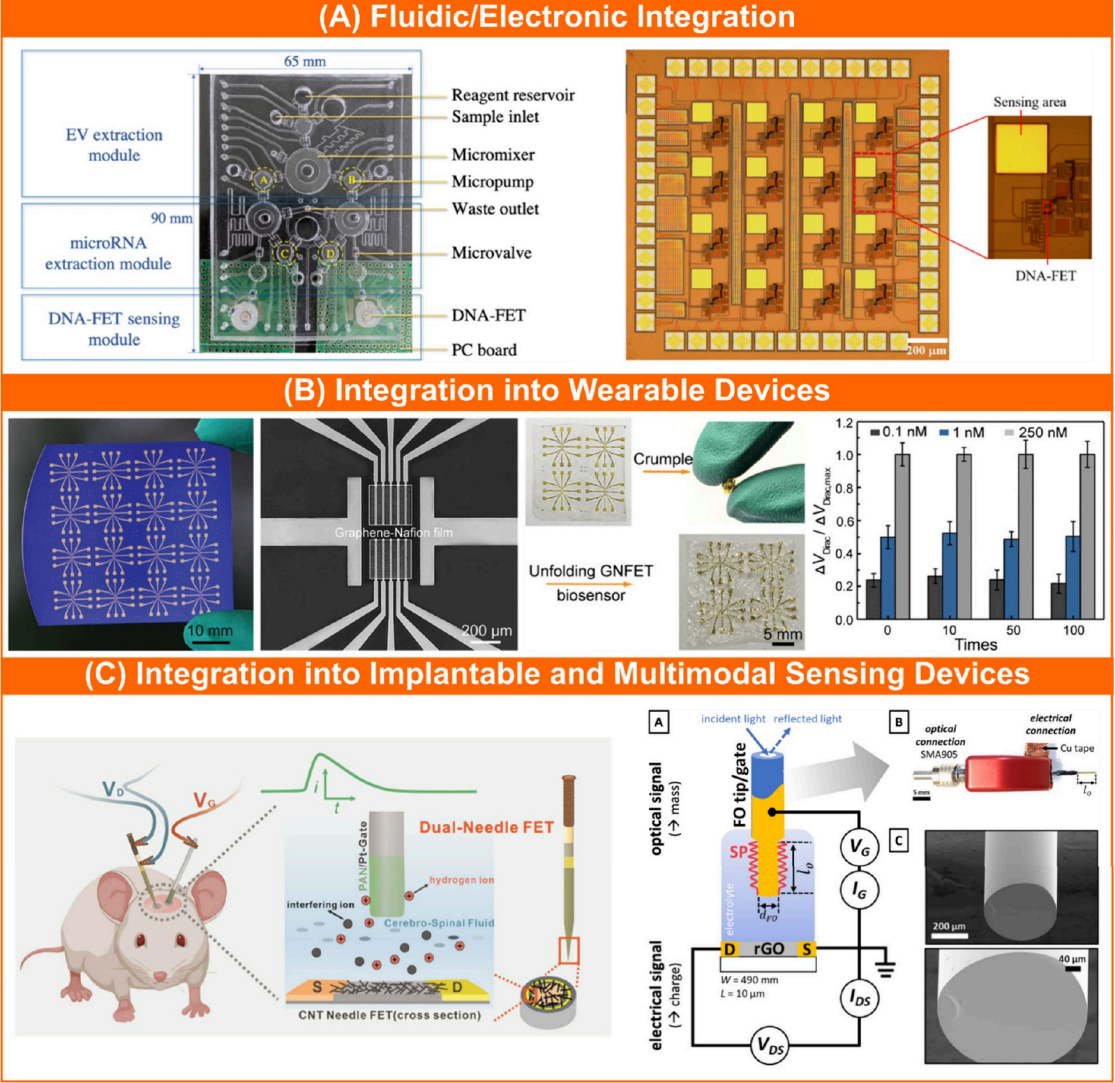
Examples of hybrid fluidic/electronic and other emerging integration formats. (A) Advanced synergy of electronic and fluidic integration in the FET-based sensing system for the detection of two breast cancer biomarkers (microRNA-195 and microRNA-126). Reproduced with permission from ref (90). Copyright 2021 Springer Nature. (B) Highly deformable and regenerative composite graphene-Nafion film FET biosensor array assembled on a 6-μm thick PET substrate to detect IFN-γ in human sweat. Reproduced with permission from ref (13). Copyright 2020 Wiley. (C) Dual-needle solution-gated FET sensor design for pH monitoring in the rat brain microenvironment (left). Reproduced from ref (96). Copyright 2023 American Chemical Society. Dual-mode electro-optical electrolyte gated FET sensing system where gold-coated optical fiber simultaneously serves as the gate electrode in the reduced graphene oxide-based FET and a substrate for surface plasmon resonance spectroscopy (right). Reproduced from ref (97). Available under CC BY-NC-ND 4.0 license. Copyright 2022 Hasler et al.

Huang et al.^90^ (Figure 4A) demonstrated a device comprising a dedicated highly integrated microfluidic system interfaced with CMOS-fabricated DNA-FET sensing modules in extended gate configuration that features a specific readout strategy with frequency-based output for quantifying microRNA-195 and microRNA-126 as breast cancer biomarkers. Such a device enables quantification from 100 μL of undiluted plasma with detection limits of 84 aM (microRNA-195) and 75 aM (microRNA-126) within 5 h by using an automated multistep process that comprises: 1) extraction of extracellular vesicles (EVs) by anti-CD63 beads with 83.5% efficiency, 2) isolation of microRNAs from EVs through hybridization relying on magnetic beads functionalized by amine-modified cDNA probes with the efficiencies of 85.4% (microRNA-195) and 93.5% (microRNA-126), and 3) quantification of extracted microRNAs via electrical detection of the hybridization between microRNAs and thiol-modified cDNA sequences attached to the 100 μm × 100 μm gold electrodes. The setup comprised a laptop, electromagnetic valves for flow control, an air compressor, a vacuum pump, a temperature control module, a microfluidic chip, and a DNA-FET sensing module (with two biosensor chips, biasing electronics, reference electrodes, and oscilloscopes). The liquid handling was automated and pneumatically driven within an oxygen plasma-bonded three-layer microfluidic chip (PDMS-based air control and liquid channel layer and glass substrate) with a system of 3 micromixers, 7 micropumps, and 23 microvalves embedded into the air control layer. DNA-FET biosensor chips comprised 4 × 4 arrays of p-type MOSFETs with different gate widths operated in a subthreshold regime as part of the extended gate configuration. A particularly interesting integrated CMOS-based readout was incorporated into the biosensor chip that converts the electric current induced by microRNA hybridization into the shift of output signal frequency by employing a self-regulated circuit relying on controlled capacitor charging and discharging combined with voltage comparator to generate a pulsed voltage output.

Bae et al.^91^ demonstrated a standalone and power-efficient biosensing device for the multiplexed detection of SARS-CoV-2 spike proteins (LOD: 1 pg/mL) and antibodies (LOD: 200 ng/mL) featuring the integration of a 6 × 4 array of IGZO-based FET biosensors with a microfluidic system for spatial control of fluid delivery and hardware implementation of artificial neural networks for detection classification. The microfluidic guiding system composed of SU-8 and PDMS layers served to precisely deliver the fluids during bioreceptor immobilization and analyte testing, enabling minimized consumption of the analyzed specimens (<100 μL) and reagents. The FET biosensor array comprised three sets of sensing units: 1) antibody-functionalized sensors for SARS-CoV-2 spike protein detection, 2) COVID-19 spike protein-functionalized sensors for antibody detection, and 3) calibration sensors for cancelation of background interference. The biosensor array was directly interfaced with the hardware edge-computing system composed of a microcontroller and 8-channel arrays of transimpedance amplifiers and ADCs. After applying quantized gate voltages to individual FET sensors selected by a multiplexer, output current is converted to a voltage signal and digitized for further analysis using two separate three-layered (input-hidden layer-output) neural networks implemented for the direct classification of raw experimental data. The resulting classification accuracies for antibody and spike protein detection were 98.85% and 93.22%, respectively. Remarkably, the near-sensor hardware-based implementation of artificial neural networks in this case outperformed the software-based implementation by introducing just a fraction of latency (<200 μs on average) and requiring significantly lower power consumption (average of 4.22 mW).

Rothberg et al.^59^ presented an IS-FET-based and CMOS-integrated massively parallelized device for accurate and simultaneous detection of independent and spatially localized DNA sequencing reactions, thereby paving the way for cost-efficient and routine sequencing of the human genome (see Figure 2B). The device technology, commercialized by Ion Torrent^78^ (now part of Thermo Fisher Scientific Inc.), relies on the high degree of synergy between electronic integration, fluidic integration, and signal processing. Each sensor comprises a floating gate connected to an IS-FET and covered by the tantalum oxide pH sensing layer. Spatial confinement of sequencing reactions is achieved by forming wells of 3.5 μm diameter in which 2-μm-sized acrylamide beads decorated with sequencing primers and DNA polymerase are loaded by centrifugation. When a nucleotide is added to a growing DNA strand, a proton is released, changing the local pH for ca. 0.02 units in the well and causing a surface potential shift that allows detection within 4 s. Readout of individual sensors arranged in a two-dimensional array is facilitated by an in-built multiplexer relying on row and column select registers for CMOS-compatible addressing. The sensor array is robotically equipped with a disposable polycarbonate flow cell for fluidic interfacing and sample loading. The fluid delivery and transport are automated to accommodate stepwise nucleotide flow and rapid washing (within 0.1 s) after each nucleotide addition. Software for signal processing identifies incorporated nucleotides based on the raw voltage signals sampled at a high rate using a complex physical model combined with dual signal-based filtering eliminating the readouts with low accuracy. The device was fabricated with a yield of 99.9% of pH-sensitive FET-based sensors that can be packaged in arrays containing from 1.2 to 11 M IS-FETs with wells exposed to fluids.

### Critical Remarks

The development of advanced autonomous and high-precision FET sensing systems requires excellent synergy between electronic and fluidic integration. Only several demonstrations of effective synergy between electronic and fluidic integration of FET chemo- and biosensors have been reported so far. Most of these devices are still highly complex, bulky, costly, and have limited scale of electronic and fluidic integration. The biggest prospective challenges for FET sensing systems with hybrid electronic/fluidic integration are the efficient miniaturization of the device down to a portable standalone format and the specialized design of supporting electronics to satisfy the diverging requirements of FET-based sensing and accurate fluid manipulation. Advances in energy-efficient micropumping systems and precision electronics for portable instrumentation hold promise to address these challenges in the future.

## Integration of FET Sensors into Wearable and Implantable Devices

Before dealing with more complex design issues, such as integration types and levels, specific application scenarios sometimes require the consideration of basic design aspects. Among them are the shape and mechanical properties of the sensing device. One of the increasingly important requirements is to design wearable FET-based chemo- and biosensors, implicating the need to create devices that comply with the shape, texture, and mechanical properties of the skin (e.g., to endow flexibility, deformability, stretchability, etc.). Fabrication and integration on unconventional and nonplanar substrates with different levels of mechanical stiffness are also essential when designing medical sensing devices. Here, we showcase some key studies demonstrating the formation of wearable and implantable FET sensors exhibiting different levels and types of integration.

Flexible and wearable FET sensors are typically based on low-dimensional materials (predominantly graphene, CNTs, and In_2_O_3_) and produced on flexible plastic sheet substrates (100 μm or lower in thickness) compatible with common FET fabrication techniques. Such devices are commonly integrated only into small-scale arrays due to the limitations of fabrication techniques and surface area available for wearables. We also briefly mention here a few notable studies on wearable devices that rely on EG-FET architectures, showcasing the potential for simplified integration of sensing units on a larger scale.

Kim et al.^92^ demonstrated a small-scale array of coplanar graphene FETs constructed on top of flexible PET substrates modified by polystyrene brushes to prevent undesirable doping of graphene layers. The FETs were functionalized by Vesicular stomatitis Indiana virus (VSV) antibodies and the resulting FET sensors reached an LOD of 47.8 aM for VSV-enveloped model viruses (human immunodeficiency and murine leukemia viruses) in environments containing up to 50 mM NaCl. As described previously, Sun et al.^76^ fabricated an array of single-wall CNT-based FET biosensors on polyethylene naphthalate substrates modified by parylene C by fully relying on solution processing techniques (bar-coating, spin coating, screen printing, and drop casting). The fabrication process had a yield of 100% with the resulting FET devices featuring low driving voltage of up to 1 V and high field-effect mobility of 70 cm^2^/(V s), making them suitable candidates for disposable biosensors targeting negatively charged bacteria.

Wang et al.^13,93^ reported highly deformable aptamer-functionalized small-scale arrays of graphene-based FET biosensors for detecting cytokines produced on thin PET substrates (thickness <10 μm) and compatible with wearable devices. Their array of FET biosensors based on monolayered graphene channels was fabricated on a 2.5-μm thick Mylar substrate endowing high mechanical durability and consistent electrical properties even after withstanding hundreds of cycles of rolling, twisting, and stretching. The FET sensors could survive extensive deformations such as rolling on cylindric surfaces down to 40 μm radius, twisting between −180° and 180° angles, stretching up to 125%, and still successfully detect TNF-α from solution with LODs down to 5 pM. In the second realization (Figure 4B), the authors prepared flexible and regenerative FET biosensor arrays based on composite graphene-Nafion films assembled on 6-μm thick PET sheets to detect IFN-γ in undiluted biofluids (e.g., human sweat) with sub-pM LOD. The obtained highly conformal biosensor arrays could survive extensive cyclic crumpling (up to 100 cycles) without losing sensitivity and consistency of sensing response (deviation <4.1%) to IFN-γ. These sensors could also be regenerated up to 80 times using a straightforward process without substantial damage in terms of electromechanical properties.

Xu et al.^7^ demonstrated the flexible and thin FET sensor for glucose detection based on a 6 nm thick layer of In_2_O_3_ fabricated on top of a 2.9-μm thick polyimide substrate. Glucose detection relies on reversible interactions between boric-acid-containing self-assembled peptidic hydrogel and glucose that generate the modulations of boronate anion concentration and affect FET channel conductivity. Such an analyte-responsive hydrogel strategy proved useful for real-time monitoring of glucose concentration. The obtained flexible and wearable device showcased a sensitivity of 7.6 mV/dec, linearity of 93% on a logarithmic scale, and LOD for glucose in the ∼ 10 nM range. Wang et al.^5^ also reported a small array of flexible In_2_O_3_-based FET sensors capable of measuring pH (4.6–7.6) and cortisol (1 pM–1 μM) levels that are integrated into a standalone wearable device featuring the synergy of electronic and fluidic integration and enabling real-time monitoring of cortisol in sweat. They patterned 2–3 nm-thick In_2_O_3_ layers on 7-μm thick polyimide sheets, modified their surface for specific detection, and showcased that FET characteristics and sensing response remain practically unaffected after 100 cycles of bending down to the curvature radius of 15 mm. Xu et al.^94^ developed a similar sensing system for cortisol monitoring in sweat (LOD: 100 fM; range: 1 nM–100 μM) based on the extended gate configuration of a AlGaN/GaN high electron mobility transistor in the format of a wearable sticker.

Lefler et al.^28^ showcased a unique FET-based sensing device with ca. 50 μm × 50 μm area that can be wafer-scale integrated, adapted for minimally invasive skin insertion and wireless signal transmission, and potentially applied for continuous monitoring of analytes in interstitial fluid. The authors implemented a system coupling an exposed, chemically inert, conductive, and polarizable working electrode with the fully passivated Si-based FinFET. Small and reversible changes in working electrode polarization resulting from the interaction with redox species present in the surrounding solution are amplified by the FET, causing the modulations of charge carrier concentration and thereby source-drain current of the FET. The response can be optimized for a specific redox analyte under physiological conditions using a voltammetric approach of fine-tuning the DC biasing potentials applied to the working electrode, source, and back gate terminals. Importantly, the designed measurement approach does not require a reference electrode. Using such a paradigm, the authors first demonstrated the in vitro sensing of H_2_O_2_ with sub-10 μM LOD. When the working electrode was functionalized with the glucose oxidase-containing hydrogel, they obtained a reliable in vitro response to glucose from 0.5 to 10 mM with an LOD of 0.25 mM.

Minimally invasive devices for real-time monitoring of pH and different ions (Na^+^, K^+^, Ca^2+^) in the interstitial fluid were also developed using the rigid templated microneedle-based EG-FET architectures.^29,95^ Devices of this kind have already reached a high level of integration and autonomous operation, encompassing a reliable and robust multianalyte monitoring (pH, Na^+^, K^+^, Ca^2+^), wireless data transmission module, and self-powering capability endowed by a hybrid powering system coupling triboelectric nanogenerator and solar cell with a rechargeable battery.^29^

Apart from minimally invasive devices that are still considered as wearables, there is a need to construct implantable devices for sensing in vivo. The highly complex biochemical environments present many challenges from the perspective of sensing stability and reliability, as well as from the perspective of biocompatibility (particularly important in monitoring applications). Only a few notable realizations focused on neuroprobes have been reported in the literature and not all of them were validated in realistic in vivo settings.

For example, Zhao et al.^30^ demonstrated flexible and implantable aptamer-functionalized neuroprobes for the detection of serotonin (pM−μM range) in artificial cerebrospinal fluid with high ionic strength that are placed inside a brain hydrogel phantom. The probes were fabricated on a 7-μm thick polyimide substrate with a width of 150 μm and two FET sensors based on a 3 nm thick In_2_O_3_ semiconducting film situated near the tip. 150 of such probes could be scalably produced on a 4 in. Si wafer. The probes were implanted using 150-μm thick supporting Si substrates together with Ag/AgCl wire reference electrodes within ∼ 1 min into the brain-mimicking hydrogel (0.6% w/v of agarose in artificial cerebrospinal fluid) that was subsequently flushed with serotonin for measurements. The implanted flexible neuroprobes effectively detected the diffusion of serotonin from the injection site to the probe location (∼2 mm distance).

Interesting implantable neuroprobes based on FET sensors have been constructed using the acupuncture needle as a base material.^31,96^ Zhou et al.^31^ showcased the needle-based FET sensor with an aptamer-functionalized reduced graphene oxide channel, that is capable of real-time in vivo monitoring in the rat brain. The real-time in vivo monitoring was shown for dopamine and neuropeptide Y under the influence of stimulants and drugs, but the proof-of-concept was illustrated also for other targets, such as transmembrane glycoprotein Mucin 1 and miRNA21. The FET sensor was constructed by an alternated coating of Au and parylene layers to create a coaxial sandwich structure. This was followed by drop casting of reduced graphene oxide to connect the device terminals (gold source and stainless-steel drain) and prepare for further tailored surface modification. The resulting device was portable, mechanically rigid, and reusable after the regeneration process comprising grinding and ultrasonic cleaning. However, such devices still feature batch-to-batch variations due to challenging functionalization and require independent implantation of the Ag/AgCl reference electrode. Liu et al.^96^ (Figure 4C, left) demonstrated a more advanced dual-needle solution-gated FET sensor design for pH monitoring in the rat brain microenvironment. The sensor showed a highly stable, reliable, selective, and sensitive (53.7 mV/pH) response to dynamic pH changes in the cerebrospinal fluid of the living rat brain within the wide range of pH from 4 to 9. The first needle-based part was constructed similarly as in the work of Zhou et al.^31^ only using high-purity CNTs as the semiconducting channel material, while the second “needle” played the role of a pH-sensitive gate composed of a Pt wire electrochemically functionalized with a layer of polyaniline. Although biocompatible, the needle-based FET sensing systems are not suitable for long-term monitoring and present many challenges for further integration into arrays or standalone functional devices.

### Critical Remarks

There is a growing interest in designing wearable and implantable FET chemo- and biosensor devices driven by the increasing demand in the field of healthcare monitoring. Emerging low-dimensional materials and innovative fabrication approaches have enabled some successful proof-of-principle demonstrations of such devices. However, due to the highly diverse requirements in specific applications and the variety of incorporated materials, it remains challenging to define reliable and broadly applicable fabrication, integration, and interfacing strategies. Furthermore, future research should also focus on enabling FET sensor miniaturization to improve integration opportunities and designing biocompatible FET sensor devices suitable for long-term monitoring.

## Integration of FET Sensors into Multi-sensor or Multi-modal Devices

Finally, FET sensors and devices can also be integral parts of multisensor systems^3,98^ or multimodal sensing devices^97,99,100^ that do not necessarily have high integration levels but showcase improved and more comprehensive functionality, contributing to increased reliability of detection. In addition to the simplified cases of integrating a temperature sensor next to FET sensors for response monitoring and correction, interesting results can be obtained by combining FET-based approaches with some electrochemical analysis techniques and optical sensing strategies. Such multifaceted sensor systems enable the simultaneous collection of complementary data with a reduced footprint, contributing to device miniaturization and streamlined data transmission, which are key aspects for integration into digital sensing ecosystems (e.g., Internet of Things (IoT)).

Liu et al.^3^ demonstrated a flexible and multiplexed FET sensor system for simultaneous real-time monitoring of pH (range: 4–10), serotonin, and dopamine (both in the concentration range: 10 fM–1 μM) combined with a resistive temperature sensor (range: 20–50 °C). The FET sensors were based on 16 nm thick In_2_O_3_ nanoribbon channels (unmodified for pH and aptamer-modified for neurotransmitter sensing) patterned on top of a 1.4-μm thick PET substrate, allowing for robust mechanical and electrical performance even in undiluted artificial cerebrospinal fluid. The temperature sensor was fabricated by sputtering 1 nm Ti/50 nm Au layers through a shadow mask onto the PET substrate. The realized multiplexed and real-time measurement system covers remarkably wide detection ranges of all relevant markers and shows great potential for further integration into portable standalone devices. One of the important limitations of similar FET-based multisensor systems featuring diverse chemo- and biosensors is the spatial confinement of different functionalization processes. Wright et al.^101^ proposed a unique solution to this problem using thermal scanning probe lithography and a thermochemically sensitive polymer. The proposed technique allows localized immobilization of different bioreceptors down to sub-20 nm resolution and 200 nm pitch size (comparable to FET array geometries in CMOS chips), thereby unlocking the opportunities for parallel detection of numerous biomarkers by employing large-scale integration levels. Massey et al.^98^ reported the fluidic and electronic integration of two separate biosensing modules for bioanalytes in saliva and exhaled breath based on small arrays of aptamer-functionalized organic electrolyte-gated FET sensors and molecularly imprinted impedimetric sensors assembled on a common custom-built low-power PCB (<300 mW) to allow for controlled multiplexing and readout. Organic electrolyte-gated FET biosensors were fabricated within a Kapton substrate sandwich and integrated with a microfluidic channel structure to enable the detection of cortisol in saliva (range: 2.73 pM–273 μM). The impedimetric biosensor based on a molecularly imprinted polymer was prepared in the format of modified interdigitated electrodes on a silicon substrate and interfaced with an aerosolization unit for the detection of 8-isoprostane in exhaled breath. The onboard impedance analyzer showcased effective recording of the impedance profile in the frequency range 100–100 kHz with changes in geometry-based capacitance proportional to the analyte concentration.

De-Eknamkul et al.^99^ realized a dual-mode optoelectronic biosensor based on a multifunctional MoS_2_ monolayer grown by chemical vapor deposition. The device was fabricated by placing the MoS_2_ monolayer over a 100 nm thick SiN membrane containing an array of nanoholes (period of 440 nm and diameter of 120 nm) and depositing the transparent dielectric (Al_2_O_3_, 25 nm) and back gate electrode (indium tin oxide, 30 nm) layers at the bottom of the membrane. Such design allows the simultaneous creation of the slab with photonic crystal nanostructures that exhibit Fano resonances near 700 nm wavelength in the optical transmission spectra and the MoS_2_-based FET device. Accumulation of detected biomolecules at the same time causes the redshift of Fano resonance peaks due to localized changes in medium permittivity and the change in drain current of the FET due to modulations of in-plane conductivity induced by electrostatic gating. As the proof-of-principle system for biosensing, the authors used the histidine-tagged water-soluble form of the μ-opioid peptide (MOR) as a functionalized bioreceptor and synthetic target opioid peptide analog to encephalin DAMGO ([d-Ala2,N-MePhe4, Gly-ol]-enkephalin) as a bioanalyte in deionized water. In both modes, the 0.1 nM LOD was reached, and linear dependence was demonstrated in the measurement range 0.1–10 nM with the sensitivities of 10.5%/nM (relative drain current change in electronic mode), and 0.15 nm/nM (spectral displacement in optical mode).

Zhang et al.^100^ developed a dual-mode real-time biosensor enabling potentiometric and fluorescence-based readout by integrating graphene FET at the end face of the optical fiber and functionalizing the graphene channel for simultaneous optical detection based on the fluorescence resonance energy transfer (FRET). To construct the graphene FET, a graphene film grown by chemical vapor deposition was transferred to cover the source and drain contacts that were patterned as the gold film by combining magnetron sputtering and laser etching on the quartz fiber surface, and the Ag/AgCl reference electrode served as the gate. Aptamer probes modified with 6’-carboxy-fluorescein (6’-FAM) were immobilized on the graphene surface to achieve specific detection of target single-stranded DNA. To create a fluorescence biosensor based on the FRET principle, graphene oxide was added to quench 6’-FAM before the DNA analyte was introduced. This process created an optic-fiber graphene FET for the real-time detection of DNA hybridization that integrates simultaneous electronic sensing via FET drain current changes due to channel conductance modulation and fluorescence sensing based on intensity restoration due to FRET. The described sensor construction improves sensing efficiency and reliability, leading to the detection limits down to 10 nM concentration of target single-stranded DNA when a dedicated electro-optical experimental setup with a two-channel data acquisition unit is used.

Hasler et al.^97^ (Figure 4C, right) reported the dual-mode electro-optical electrolyte gated FET sensing system where gold-coated optical fiber simultaneously served as the gate electrode in the reduced graphene oxide-based FET and a substrate for surface plasmon resonance spectroscopy. This sensing principle allows for the discrimination between mass and charge contributions of bound analytes at the same surface through the electronic FET-based readout and optical readout relying on local changes of the refractive index at the sensing interface. In addition, the coupling of surface plasmons using the optical fiber facilitates the integration of sensing modalities. The authors show that in such device configuration proper positioning, sufficient length of immersed gold-coated fiber, and fiber diameter play a significant role in optimizing both types of sensing responses. The sensing performance of the system was validated with two proof-of-concept systems: 1) layer-by-layer assembly of oppositely charged polyelectrolyte multilayers (positively charged poly(diallyldimethylammonium chloride) and negatively charged poly(sodium 4-styrenesulfonate)) and 2) binding of thrombin to an immobilized biotinylated aptamer. The authors efficiently determined the average layer thickness and charge shifts correlated to layer-by-layer assembly and showcased the ability to evaluate the electrical permittivity of the assembled multilayers. Using the time-resolved titration of thrombin in the relevant range for medical applications, the authors obtained Langmuir-type saturation characteristics in both, electrical and surface plasmon resonance monitoring, allowing them to determine binding affinity constants ((2.7 ± 0.3) × 10^–9^ M from electrical and (1.4 ± 0.9) × 10^–9^ M from optical response) and LODs (0.2 nM for electrical and ∼ 1 nM for optical detection) that have comparable values.

Interestingly, apart from serving as a chemo- or biosensor, an integrated FET device can also serve as a performance enhancer in flexible optical detection systems with organic photodiodes, where it can significantly reduce the dark current and amplify the photoplethysmographic signal by a factor of 10.^102^ Similarly, the FET transducer can amplify the pH response of carbon fiber microelectrodes in artificial cerebrospinal fluid to the super-Nernstian level of sensitivity (sensitivity of 101 ± 18 mV/pH in the pH range 5–8).^103^

### Critical Remarks

Multisensor and multimodal devices incorporating FET sensors are becoming important tools for enabling reliable and diversified measurement with improved accuracy and monitoring capabilities. However, these systems are still in their infancy and many issues need to be resolved to achieve scalable fabrication and enhanced integration. Depending on the types of analytes or parameters, as well as the required diversity of incorporated sensors, multisensor systems still commonly suffer from limitations related to device-to-device variations, complexity of highly localized surface modification, and underdeveloped integration approaches. Multimodal devices face even greater integration challenges due to the highly demanding fabrication that is hard to scale up and constraining design optimization requirements accounting for different modalities in terms of material selection, geometry, sensing response, and readout methodology. Despite their current limitations, multisensor and multimodal devices show great potential for creating future systems designed to perform robust and comprehensive measurements relying on complementary detection strategies.

## Summary, Challenges, and Perspectives of FET Chemo- and Biosensor Integration

To conclude, we summarize the different integration formats and levels for FET-based chemo- and biosensor devices while highlighting the most important advances, pointing out the critical challenges, and outlining the perspectives for future research and development. A brief overview of state of the art in FET chemo- and biosensor integration is provided in Table 1. We observe that there are already significant advances in large-scale FET sensor integration when it relies on well-established Si-based materials. Conversely, the emerging low-dimensional materials still mainly show limited large-scale integration capabilities, despite a few notable results showing higher levels of scalability for carbon-based materials such as graphene and CNTs. The main reasons for this are insufficient compatibility with established highly scalable FET fabrication strategies inherited from microelectronics and a lack of effective alternative fabrication approaches. With the increasing demands of circular economy and personalized wearable devices, flexible, recyclable, and degradable materials coupled with cost-effective fabrication will become crucial. Promising results have been shown for In_2_O_3_-based FETs that can be efficiently produced in a scalable manner using simplified processes even on thin and highly deformable polymeric substrates. Fabrication strategies based on solution processing (e.g., different printing methods) hold promise for advancing and revolutionizing FET sensor integration. One of the key fabrication challenges that remains insufficiently addressed is the scalable and reproducible formation of FET sensors directly on curved rigid substrates, a challenge important for certain biomedical applications involving implantable devices.

**Table 1:** Brief Overview of State of the Art in FET Chemo- and Biosensor Integration

Biosensor architecture	Sensing interface	Number of sensing units	Integration format	Standalone device?	Target analytes	LOD	Sensitivity	Application	Ref.
Graphene-based FET	Aptamer-modified graphene FET employing buried-gate geometry with HfO_2_ dielectric	1	Electronic (basic)	Yes	Interleukin-6	12 pM	∼25%/nM^a^	Measurement of cytokines in saliva	Hao et al. 2019^53^
MoS_2_ nanosheets-based FET sensor array	Antibody-functionalized MoS_2_ nanosheets	4	Electronic (SSI)	No	Nuclear matrix protein 22 (NMP22); cytokeratin 8 (CK8)	0.027 aM; 0.019 aM	0.12 μA/dec; 0.1 μA/dec^a^	Diagnosis of bladder cancer	Yang et al. 2020^11^
Array of semiconducting single-wall CNT FETs	Film of semiconducting single-wall CNTs	>100 (scalable)^a^	Electronic (MSI)	No	*Shewanella onedensis* MR-1	10 CFU or 10^5^ CFU/mL	14%/dec	Detection of bacteria	Sun et al. 2023^76^
SiNW Schottky-junction FET array	Aptamer-functionalized HfO_2_ shell of SiNWs	1024 (32 × 32)	Electronic (LSI)	Yes	Dopamine	∼fM	Up to ∼1 V/fM	Neurotransmitter level monitoring	Sessi et al., 2022^2^
Dual-gated BioFET array	HfO_2_ sensing layer	1048576 (1024 × 1024 array)	Electronic (ULSI)	No	pH	Range 4–10	Up to 84 mV/pH	Highly reliable pH measurement	Duarte-Guevara et al. 2017^26^
Single-crystal graphene-based FET array	Protein kinase Abl1-functionalized graphene	7^a^	Fluidic (basic) + electronic (SSI)	No	Imatinib	15.5 fM	∼0.0194 μA/fM	Analysis of drug binding affinity and kinetics	Xu et al. 2021^6^
Floating gate CNT FET covered by Y_2_O_3_/HfO_2_ array	CNT channel layer coated with Y_2_O_3_/HfO_2_	>700 at wafer scale (17 in a cluster)^a^	Fluidic (basic) + electronic (MSI)	No	pH	Range 1.34–12.68	67.62 mV/pH	Continuous pH monitoring with low hysteresis	Wang et al. 2023^65^
Single-layer graphene FET array	Single-layer graphene FET modified by enzyme-encoded hydrogel stamp	7^a^	Fluidic (basic) + electronic (SSI)	No	Penicillin; urea	0.25 mM; 1 mM	∼12 mV/mM^a^; ∼7 mV/mM^a^	Design and testing of a modularized FET biosensor	Dai et al. 2019^71^
Graphene micropattern FET array	Antibiotics-conjugated graphene micropattern FETs	2^a^	Fluidic (basic) + electronic (SSI)	Yes	*Escherichia coli*; *Salmonella enterica*; *Staphylococcus aureus*; *Enterococcus faecium*	1–9 CFU/mL	0.03–0.33%/CFU^a^	Real-time detection and Gram-typing of bacteria	Kim et al. 2020^82^
Graphene FET with metallic split-float-gate	Antibody-functionalized Au surface of the split-float-gate	3	Fluidic (medium) + electronic (SSI)	No	Carcinoembryonic antigen (CEA); α-fetoprotein (AFP); parathyroid hormone (PTH)	10.09 nM; 22.06 nM; 9.87 nM	Not quantifiable	Liver cancer diagnostics and screening	Wang et al. 2024^83^
SiNW-based FET	*Mycobacterium tuberculosis* Ag85B antibody-modified SiNW-based FET	1	Fluidic (complex) + electronic (basic)	Yes	*Mycobacterium tuberculosis*	1.0 fg/mL	0.52/dec (normalized voltage response)	Detection of bacteria	Xie et al. 2022^87^
Si-based p-type MOSFET array	DNA-probe modified gate of the p-type MOSFET	16	Fluidic (complex) + electronic (SSI)	Yes	microRNA-195; microRNA-126	84 aM; 75 aM	3.4 Hz/fM; 3.3 Hz/fM	Detection of breast cancer biomarkers	Huang et al. 2021^90^
IGZO-based FET array	SARS-CoV-2 spike protein and antibody-modified IGZO-based FET	24 (6 × 4)	Fluidic (medium) + electronic (SSI)	Yes	SARS-CoV-2 spike protein; SARS-CoV-2 antibody	1 pg/mL; 200 ng/mL	mainly classification tool	Detection of COVID-19 biomarkers	Bae et al. 2023^91^
Array of IS FETs based on Ta_2_O_5_ layer	Ta_2_O_5_ pH sensing layer + acrylamide microbeads decorated with sequencing primers and DNA polymerase	Up to 11M^a^	Fluidic (complex) + electronic (ULSI or SLSI)	Yes (commercial)	DNA sequence	Single nucleotide	∼0.02 pH/nucleotide (or ∼1.16 mV/nucleotide)	Large-scale and accurate detection of DNA sequencing reactions	Rothberg et al. 2011^59^
Graphene-Nafion film-based FET array	Aptamer-functionalized graphene-Nafion composite film	16^a^	Electronic (SSI) + flexible device	No	IFN-γ	740 fM	20%/dec^a^ (relative voltage shift)	Detection of cytokines in sweat	Wang et al. 2021^13^
In_2_O_3_-based FET array	Peptidic hydrogel gated In_2_O_3_ FET	8^a^	Electronic (SSI) + flexible device	No	Glucose	∼10 nM	7.6 mV/dec	Real-time monitoring of glucose	Xu et al. 2023^7^
Polarizable working electrode with the fully passivated Si-based fin-shaped FET array	Working electrode (bare or coated with enzyme-containing hydrogel)	4 per device^a^	Electronic (SSI) + wearable device	Yes	H_2_O_2_; glucose	∼10 μM; 0.25 mM	Not quantifiable	Continuous glucose monitoring	Lefler et al. 2022^28^
Microneedle-based EG-FET arrays	Microneedle arrays modified by ion-sensitive membranes and polyaniline	4 per device^a^	Electronic (SSI) + wearable device	Yes	pH; Ca^2+^; Na^+^; K^+^	Range 5–9; 30.96 μM; 0.56 μM; 24.23 μM	51.77 mV/pH; 20.11 mV/dec; 104.33 mV/dec; 43.42 mV/dec	Monitoring of biomarkers in interstitial fluid	Omar et al. 2024^29^
In_2_O_3_ thin film FET array	Aptamer-modified In_2_O_3_ film	2 per neuroprobe^a^	Electronic (SSI) + implantable device	No	serotonin	∼pM	∼1.6 mV/dec^a^	Neurotransmitter monitoring	Zhao et al. 2022^30^
Acupuncture needle-mounted reduced graphene oxide-based FET	Aptamer-functionalized reduced graphene oxide channel	1	Electronic (basic) + implantable device	No	Dopamine; neuropeptide Y; transmembrane glycoprotein Mucin 1; miRNA21	∼1 nM; ∼0.1 nM; ∼0.1 pg/mL; ∼0.1 pM	∼20 mV/dec^a^; ∼10 mV/dec^a^; ∼20 mV/dec^a^; ∼20 mV/dec^a^	Real-time monitoring of neurotransmitters in the brain	Zhou et al. 2022^31^
Dual-needle solution-gated CNT-based FET	High-purity CNT-based semiconducting channel + polyaniline-functionalized Pt wire	1	Electronic (basic) + implantable device	No	pH	Range 4–9	53.7 mV/pH	pH monitoring in the brain	Liu et al. 2023^96^
Array of In_2_O_3_ nanoribbon FETs	Unfunctionalized and aptamer-functionalized In_2_O_3_ nanoribbons	56 (14 × 4 array)	Multisensor + electronic (MSI) + flexible device	No	Serotonin; dopamine; pH; temperature	10 fM; 10 fM; range 4–10; range 20–50 °C	0.05 I_0_/dec; 0.044 I_0_/dec; 1.25 I_0_/dec^a^; 3.9 Ω/°C	Monitoring of chemical processes in the brain	Liu et al. 2020^3^
Dual-mode optoelectronic biosensor based on a multifunctional MoS_2_ monolayer	Photonic crystal nanostructures + MoS_2_-based FET	1	Multimodal + electronic (basic)	No	DAMGO	0.1 nM (electronic and optical mode)	10.5%/nM (electronic) and 0.15 nm/nM (optical)	μ-opioid peptide detection	De-Eknamkul et al. 2019^99^
Dual-mode optical fiber-graphene FET potentiometric and fluorescence sensor	Graphene film with dedicated aptamer-fluorescein-based surface modification	1	Multimodal + electronic (basic)	No	Target single-stranded DNA	10 nM	Not quantifiable	Real-time detection of DNA hybridization	Zhang et al. 2021^100^
Dual-mode electro-plasmonic electrolyte gated reduced graphene oxide-based FET sensor with gold-coated optical fiber gate and substrate	Biotinylated aptamer-modified reduced graphene oxide layer	1	Multimodal + electronic (basic)	No	Thrombin	0.2 nM (electronic detection); ∼1 nM (plasmonic detection)	Not quantifiable	Time-resolved detection of thrombin	Hasler et al. 2022^97^

a Estimated values based on the data presented in the source publication.

Many of the studies demonstrating FET sensor integration are limited to the formation of FET arrays and do not address the issue of forming standalone devices incorporating the capabilities for addressing sensing units, facile data transmission, and miniaturized electronic readout. The beneficial synergies between electronic and fluidic integration also remain vastly underexplored and should be a significant topic for future research. The biggest integration challenges likely appear in multimodal sensing where the material, geometry, and readout incompatibilities (e.g., between electronic and optical systems) limit the opportunities for efficient and scalable production.

Although increased levels of integration promise to enhance many features of the FET-based measurement systems, advanced integration can also emphasize current issues and introduce new demands related to FET sensor design. Current FET chemo- and biosensors development still faces major challenges that often impede the transition between laboratory-scale prototypes and commercial sensors suitable for scalable production. Fabrication of FET sensor arrays and assemblies is still hampered by significant variations in device characteristics and the reproducible performance of detection layers commonly remains hard to achieve. These fabrication limitations become more pronounced for large-scale FET sensor arrays and limit scalability. High levels of integration are also coupled with the added complexity of the electronic measurement system in terms of hardware interfacing, signal processing, and the formation of standalone sensing devices.

Up to this point, very few devices relying on FET chemo- and biosensors have been successfully commercialized and retained on the market. These devices rely on the well-established IS-FET detection principle. In addition to the already described Ion Torrent^78^ DNA sequencing system, other devices based on the IS-FET sensor architecture enable robust pH measurements in diverse aqueous environments^104−107^ or selective detection of some small ions (e.g., K^+^ and NO_3_^–^) via ion-selective membranes.^107^ The key reasons for inefficient commercialization can be found in the limited accuracy, stability, and reliability of FET sensors that are necessary in many application scenarios. Effective synergies of different integration formats can contribute to improved cross-validation and more reproducible measuring conditions while further scaling of FET arrays can produce sufficiently robust statistics, reference measurements, and redundancy to overcome these issues. Notably, an emerging approach of using hardware-implemented real-time processing based on artificial intelligence algorithms also shows great promise for improving the reliability of FET-based sensing systems. Due to the complexity and diversity of the challenges involved in FET sensor integration, these should be addressed by multidisciplinary research efforts of scientists, engineers, and future users of advanced FET-based chemo- and biosensing devices.

Despite various challenges that currently hamper the efficient commercialization of FET chemo- and biosensors, these devices with different integration levels (from SSI to SLSI) are still expected to make transformative contributions, especially in the biomedical field. The devices reaching SSI and MSI levels have strong potential for use in clinical PoC diagnostics and effective incorporation into the digital health ecosystem (as part of the IoMT concepts). The devices featuring different levels of LSI hold promise to become powerful tools for comprehensive in vitro biomarker screening or robust in vivo mapping of healthcare parameters with high spatiotemporal resolution.

## Abbreviations

FET: field-effect transistor
PoC: point of care
EG: extended gate
IoT: Internet of Things
IoMT: Internet of Medical Things
SiNW: Si nanowire
CMOS: complementary metal-oxide semiconductor
LOD: limit of detection
PET: polyethylene terephthalate
DNA: deoxyribonucleic acid
USB: Universal Serial Bus
MOSFET: metal-oxide-semiconductor field-effect transistor
SSI: Small-Scale Integration
MSI: Medium-Scale Integration
LSI: Large-Scale Integration
VLSI: Very Large-Scale Integration
ULSI: Ultra Large-Scale Integration
SLSI: Super Large-Scale Integration
ADC: analog-to-digital converter
ELISA: enzyme-linked immunosorbent assay
PCR: polymerase chain reaction
CNT: carbon nanotube
IGZO: indium gallium zinc oxide
PCB: printed circuit board
PBS: phosphate-buffered saline
RNA: ribonucleic acid
CRISPR: clustered regularly interspaced short palindromic repeats
3D: three-dimensional
IS: ion-sensitive
TSMC: Taiwan Semiconductor Manufacturing Company
IC: integrated circuit
PDMS: polydimethylsiloxane
PMMA: poly(methyl methacrylate)
UV: ultraviolet
UHF: ultrahigh frequency
RF: radio frequency
EV: extracellular vesicle
VSV: Vesicular stomatitis Indiana virus
FRET: fluorescence resonance energy transfer.

## Vocabulary

Field-effect transistor (FET): An electronic device with three terminals (gate, source, and drain) that relies on the use of an electric field applied through the gate to modulate the conductivity of the semiconducting channel between the source and drain, and consequently the current flowing between these terminals.
Chemosensor: Any chemical sensor apart from those detecting biomolecules and biological entities. A chemical sensor is a device that transforms chemical information (e.g., analyte concentration or chemical composition of the sample) via the molecular recognition system and physicochemical transducer into a measurable signal that can be used for analysis.
Biosensor: A chemical sensor that employs a molecular recognition system based on specific biochemical or other mechanisms to detect biomolecules and biological entities.
Transducer: A part of the sensor that enables the conversion and transfer of the sensing signal from the native domain of the recognition system to the suitable output signal domain for measurement (commonly electrical or optical).
Integration: Incorporation of sensors into larger systems or devices by design to achieve specific functionality or enable synergistic effects leading to improved operation, performance, and reliability.
Multimodal sensing device: A sensing device used to simultaneously measure and process different forms of energy (e.g., electrical and optical) using the same or separate sensor units.
Multisensor system: A functional assembly of multiple sensors that detect different target analytes using equivalent or distinct sensor modalities.
